# Patient and Public Perceptions in Canada About Decentralized and Hybrid Clinical Trials: “It’s About Time we Bring Trials to People”

**DOI:** 10.1007/s43441-024-00665-y

**Published:** 2024-06-21

**Authors:** Dawn P. Richards, John Queenan, Linnea Aasen-Johnston, Heather Douglas, Terry Hawrysh, Michael Lapenna, Donna Lillie, Emily I. McIntosh, Jenna Shea, Maureen Smith, Susan Marlin

**Affiliations:** 1 Clinical Trials Ontario, MaRS Centre, West Tower, 661 University Avenue, Suite 460, M5G 1M1 Toronto, ON Canada; 2 Department of Public Health Sciences, Queen’s University, K7L 3N6 Kingston, ON Canada; 3 BESPOKE Business Solutions Inc, 135 Coons Road, L4E 2M9 Richmond Hill, ON Canada; 4 College of Lived Experience Member, Clinical Trials Ontario, MaRS Centre, West Tower, 661 University Avenue, Suite 460, M5G 1M1 Toronto, ON Canada

**Keywords:** Decentralized clinical trials, Hybrid clinical trials, Public perceptions, Public perspectives, Patient Perceptions, Patient Perspectives, Survey

## Abstract

**Background:**

Little is known about patient and the public perspectives on decentralized and hybrid clinical trials in Canada.

**Methods:**

We conducted an online survey (English and French) promoted on social media to understand perspectives of people in Canada about decentralized and hybrid clinical trials. The survey had two sections. We co-produced this project entirely with patient, caregiver, and family partners.

**Results:**

The survey had 284 (14 French) individuals who started or completed Section 1, and 180 (16 French) individuals who started or completed Section 2. People prefer to have options to participate in clinical trials where aspects are decentralized or hybridized. 79% of respondents preferred to have options related to study visits. There were concerns about handling adverse events or potential complications in decentralized trials, however, communication options such as a dedicated contact person for participants was deemed helpful. Most respondents were amenable to informed consent being done at a satellite site closer to home or via technology and were split on privacy concerns about this. Most preferred travel to a site within an hour, depending on what the trial was for or its impact on quality of life. Due to the response rate, we were unable to explore associations with gender, age, health status, geography, ethnicity, and prior clinical trial participation.

**Conclusion:**

Our findings indicate an openness in Canada to participating in trials that decentralize or hybridize some aspects. These trials are perceived to provide benefits to participants and ways to increase equity and accessibility for participants.

**Supplementary Information:**

The online version contains supplementary material available at 10.1007/s43441-024-00665-y.

## Introduction

In the past decade, there has been increasing discussion in the literature about creating and delivering clinical trials that are more ‘patient-centered’ [1–4]. One approach to this may be designing and conducting clinical trials that are decentralized or hybrid in nature. As defined by Health Canada, decentralized trials (also called remote or virtual clinical trials) are “…clinical trials conducted with study participants located outside of clinical research centres for all of the required trial procedures” [5]. We refer to hybrid trials as being those that are not completely decentralized in nature. Necessitated by safety for participants at the start of the COVID-19 pandemic, many clinical trials pivoted to decentralized or hybrid models [6–8]. Decentralized and hybrid clinical trials may also be part of a larger strategy to ensure greater equity, diversity and inclusion with respect to participation [9, 10].

Our study aimed to assess the attitudes of potential participants towards decentralized and hybrid clinical trials, and to explore associations with gender, age, health status, geography, ethnicity, and prior clinical trial participation. There are reports in the literature of the perspectives of trial staff (clinical, research, management, administration, technology and data), vendors and patient representatives [11, 12], rare disease families/individuals [13], oncology patients (in the US and the UK) [12], community members (of the organization James Lind Care in UK and Denmark) [14] and some reports from general participants (global but mostly US-based) [15] about decentralized and hybrid clinical trials. However there has been no research published to date on the perceptions and attitudes of potential clinical trial participants in Canada specifically with respect to decentralized or hybrid trials. As Clinical Trials Ontario (CTO) seeks to help improve the environment in Ontario (and beyond) to perform clinical trials [16], we felt that this important missing perspective should be further explored. Our aim is to contribute to knowledge in this area and to ensure these perspectives are heard through creating tools and resources that may be of benefit to the clinical trials community.

This work is the result of a collaboration with members of CTO’s College of Lived Experience (“The College”) [17]. College members are individuals who are patient, family or caregiver partners and who live in Ontario. College members have different lived health, health care and clinical trials experiences and bring these lived experiences to their work with CTO in various ways. In this paper, we will highlight their contributions in each section given how instrumental they were to all aspects of this work.

## Materials & Methods

This was a descriptive, cross-sectional, self-reported, open survey. The Checklist for Reporting Results of Internet E-Surveys (CHERRIES) is filled out and available in Supplementary Materials [18]. The survey was administered via the Qualtrics online survey data collection platform. Respondents were a convenience sample of people invited to participate in this web-based survey via a social media advertising campaign: CTO and CTO’s partner organizations (patient organizations and health charities) posted recruitment messages via Twitter (now X) and Facebook and embedded these messages in organizational newsletters where available. Participant recruitment followed a modification of Dillman’s Total Design Method [19]. Social media posts were made at 3 time points after the original posting: at 1 week, at 6 weeks and at 12 weeks. Recruitment and data collection occurred between September 19, 2022, and December 31, 2022.

### Sample Size

We estimated that approximately 5000 potential participants would see the invitation to participate, based on the survey being distributed through various mailing lists and on social media, and that between 21% and 72% would fill out the survey questionnaire [20]. A minimum sample size of 385 was calculated to have a confidence level of 95% with a 5% margin of error [21].

### Study Survey and Variables

The Center for the Information and Study of Clinical Research Participation’s (CISCRP’s) annual Perceptions and Insights Study survey (2021) provided a useful starting framework for questions and the survey was finalized based on input and guidance from CTO’s College of Lived Experience [15]. The study survey was divided into two sections. Section 1 consisted of 39 questions that covered individuals’ attitudes and preferences about participating in centralized and decentralized clinical trials, their health status, and demographics. Section 2 was optional and consisted of a further 42 questions that covered issues related to: informed consent, trial related travel, data collection, keeping a patient diary, home delivery of study materials, and accessibility. Questions in each section included different response options such as: 5-point Likert scales, multiple choice, ranked, or open-ended text. Each section was designed to take approximately 10 min to complete. Section 1 consisted of 1 to 2 items per page over 20 pages and Sect. 2 consisted of 1 to 2 items per page over a further 25 pages, for a total of 45 pages. The survey was available in both of Canada’s official languages, English and French. French language responses were translated and combined with the English language responses. The full survey is found in Supplementary Materials.

### Informed Consent, Data Integrity and Storage

Informed consent was obtained from each survey participant. Participants were informed about the study background, purpose, its voluntary nature, expected time to completion, and that no directly identifiable information would be collected or reported, including IP addresses. We conducted a manual review of the responses and found no evidence of multiple entries made by single individuals. Participants were informed that the study data would be stored on an encrypted hard drive on Queen’s University servers and kept securely for at least five years as per Queen’s University policy [22, 23], after which the study data will be deleted. Only two study team members (JQ, SM) have access to the survey data. The study was approved by the Queen’s University Health Sciences and Affiliated Teaching Hospitals Research Ethics Board.

### Data Analysis

Frequency counts and percentages for all the study variables were tabulated and calculated. Open-ended text responses were processed into categories via content analysis by two members of the team (JQ, DPR) until consensus was achieved. All available responses were analyzed.

### Patient and Public Engagement

CTO’s College of Lived Experience members were instrumental in generating the idea for this survey, in designing and launching the survey, and in analyzing and interpreting the survey results. Our engagement approach included several virtual workshops (using Zoom) over approximately one year to understand if such a survey would be of value, to co-design the survey questions, and to review and analyze survey results so as to understand different lived perspectives related to these. Members who were interested in participating in the project or in different aspects of the project were asked to register for each virtual meeting. An application called Basecamp was used to communicate privately with College members instead of email. Any materials or expectations related to meetings were provided about one week in advance of the meetings along with details about an honorarium that was offered and how to accept it. Generally, two meetings with the same agenda were hosted on different dates and times to enable more members to attend and each meeting was hosted as a discussion where notes were taken and members’ input was used to shape all aspects of the project. For example, a number of meetings were hosted to determine the exact wording of survey questions and responses. College members pre-tested the survey before it was launched. A number of College members (HD, TH, DL, ML, EM, JS, MS) are authors on this paper or have been acknowledged for their contributions (MJ, JD). We have included the Guidance for Reporting Involvement of Patients and the Public (GRIPP2) checklist to detail College members’ involvement in this work in Supplementary Materials [24].

## Results

### Participants

A total of 325 people consented to participate (23 French language) and 2 formally declined consent (the overall completion rate was 0.99). Two hundred and eighty-four participated in Sect. 1 (14 French language) and 180 participated in Section 2 (6 French language). Of the 284 who participated in Section 1, 248 completed the last closed ended question (for a completion rate of 0.87). Of the 180 who participated in Section. 2, 169 completed the last closed ended question (for a completion rate of 0.94).

#### Demographics and Perceived Health Status

Table 1 presents the respondents’ demographic characteristics and perceived health status.

**Table 1:** Demographic characteristics and perceived health status of survey respondents

Demographic characteristic	Response
Age (in years)	
	Mean = 51.0 (SD = 14.4, Range = 20–83)
**Age Category**	
20–34	36 (16.3%)
35–49	64 (29.0%)
50–64	78 (35.3%)
>=65	43 (19.5%)
**Gender**	
Woman	183 (76.6%)
Man	50 (20.9%)
Gender fluid, non-binary, and/or Two-Spirit	4 (1.7%)
Prefer not to answer	2 (0.8%)
**Highest Education**	
High school or less	2 (0.8%)
Some college or trade school or university	14 (5.8%)
Completed college or trade school certificate or diploma	31 (12.9%)
University Undergraduate	72 (29.5%)
University Graduate Level/Postgraduate/Professional	118 (49.0%)
Prefer not to answer	4 (1.7%)
**Ethnicity**	
White/Caucasian	202 (84.9%)
Chinese	8 (3.4%)
South Asian	7 (2.9%)
Black	8 (3.4%)
Southeast Asian	5 (2.1%)
Other	20 (3.3%)
**Current Daily Life Status**	
Working full time	108 (45.2%)
Retired	55 (23.0%)
Long-term disability	31 (13.0%)
Working part-time	19 (7.9%)
Other unpaid	26 (10.9%)
**Household Income**	
less than $15,000	9 (3.8%)
$15-$29,000	5 (2.1%)
$30 -$49,000	18 (7.7%)
$50-$74,000	37 (15.7%)
$75-$99,000	37 (15.7%)
more than $100,000	92 (39.1%)
Prefer not to answer	37 (15.7%)
**Urban/Rural status**	
Urban	201 (91.4%)
Rural	19 (8.6%)
Geographical location*	
West Coast	29 (12.7%)
The North	2 (1.0%)
The Prairies	26 (11.5%)
Central	154 (67.8%)
Atlantic	16 (7.0%)
**Health Status Question**	**Response**
**How would you consider your health?**	
Very poor	4 (1.6%)
Poor	27 (11.0%)
Fair	70 (28.5%)
Good	98 (39.8%)
Excellent	47 (19.1%)
**Do you live with a chronic health condition, and if so, how many?**	
None	78 (31.8%)
1	82 (33.5%)
2	42 (17.1%)
3 or more	43 (17.6%)
**Do you have a life-threatening illness now or have you in the past?**	
Yes	85 (35.6%)
No	151 (61.1%)
Unsure	8 (3.2%)

* West Coast (British Columbia), The North (Yukon, Northwest Territories, Nunavut), The Prairies (Alberta, Saskatchewan, Manitoba), Central (Ontario, Quebec), Atlantic (Newfoundland and Labrador, New Brunswick, Prince Edward Island, Nova Scotia)

Fifty-one (51) years was the average age, the youngest being 20 years and the eldest being 83 years. The majority of participants identified as a woman (76%), most were university educated (79%) and fewer than half reported that they worked full-time (45%). Very few of the study participants identified themselves as non-Caucasian (8%) and most lived in an urban area (91%). Most of the respondents were from Ontario and Quebec, which are Canada’s two most populous provinces (68%). Fifty-nine percent (59%) of respondents considered their health to be either good or excellent, 68% reported that they had at least one chronic condition (defined as a condition that lasts for 1 year or more and needs ongoing medical care or limits activities of daily living, or both) and 36% reported that they had a life-threatening illness.

#### Clinical Trial Participation, Knowledge, Preferences, and Perceptions

Table 2 presents the respondents’ clinical trial participation, knowledge, preferences, and perceptions. Close to 40% of the respondents indicated that they had participated in a clinical trial and 29% indicated that someone they cared for had participated in a clinical trial. Close to 70% of the respondents reported that they felt informed or very informed about their knowledge of clinical trials.

**Table 2:** Clinical trial participation, knowledge, preferences, and perceptions

Question and Response Options	Responses
**Have you ever participated in a clinical trial?**	
Yes	98 (39.5%)
No	141 (56.9%)
Unsure	9 (3.6%)
**Has someone you care for ever participated in a clinical trial (e.g., a spouse, child, parent, etc.)?**	
Yes	72 (29.0%)
No	155 (62.5%)
Unsure	21 (8.5%)
**Overall, how would you rate your knowledge of clinical trials?**	
Not at all informed	6 (2.4%)
Somewhat informed	21 (8.5%)
Partially informed	48 (19.4%)
Informed	102 (40.2%)
Very informed	71 (28.6%)
**When thinking about the different ways you could participate in a clinical trial, how important is it to you to be provided with options for where to have your study visits (for example, study visits at a clinical trial site, virtual or in-person visits at home, etc.)?**	
Extremely important	84 (29.6%)
Very important	137 (49.3%)
Moderately important	41 (15.8%)
Slightly important	11 (3.9%)
Not at all important	7 (2.5%)
**I would prefer to have all my study visits at the study clinic and seeing the study doctor and staff in-person at the clinic only.**	
Strongly agree	22 (7.9%)
Somewhat agree	43 (15.5%)
Neither agree nor disagree	67 (24.1%)
Somewhat disagree	90 (32.4%)
Strongly disagree	56 (20.1%)
**I would prefer to have some study visits at my home and some visits at the study clinic and seeing the study doctor sometimes via video conference from home or in-person at the clinic.**	
Strongly agree	91 (33.1%)
Somewhat agree	124 (46.2%)
Neither agree nor disagree	40 (14.5%)
Somewhat disagree	14 (5.1%)
Strongly disagree	6 (2.2%)
**I would prefer having a nurse or study team member come to my home for all my study visits and seeing the study doctors via video conference from home.**	
Strongly agree	30 (11.3%)
Somewhat agree	80 (30.2%)
Neither agree nor disagree	66 (24.9%)
Somewhat disagree	65 (24.5%)
Strongly disagree	24 (9.1%)
**I would prefer to collect all my health data at home using technology and only talking to the study team via video conference from home.**	
Strongly agree	39 (14.7%)
Somewhat agree	96 (36.1%)
Neither agree nor disagree	56 (21.1%)
Somewhat disagree	59 (22.2%)
Strongly disagree	16 (6.0%)

When asked to rate how important it was to be provided options for where to have their study visits, 79% of the respondents noted that it was very or extremely important to them. When asked to rate their level of agreement/disagreement with several statements related to their preferences; 23% somewhat or strongly agreed that it was important to have all study visits at the study clinic only, 79% somewhat or strongly agreed that it was important to have some visits at the study clinic and some visits via video conference or at their home, 42% somewhat or strongly agreed that they would prefer having a nurse or team member visit their home and see study doctors via video conference from home, and 51% somewhat or strongly agreed that they would prefer to collect all their health data at home using technology and only talking to the study team via video conference from their home.

#### Participating in a Decentralized Trial

Table 3 presents the respondents’ communication preferences, concerns, and perceptions of the potential benefits of participating in a decentralized clinical trial.

When asked to rank what types of communication with a trial team would be most helpful to them, having one person as a “go to” contact was the most highly ranked followed by: access to 24-hour help line, instructions of what to do in an “off hours” emergency, the ability to send a text message and, access to an information hub.

When asked about their level of concern about several selected elements related to participating in a decentralized trial; 64% were somewhat to extremely concerned about how to handle adverse events, 66% were somewhat to extremely concerned about how to handle potential complications, 39% were somewhat to extremely concerned about the overall quality of the research, 28% were somewhat to extremely concerned about using or accessing technology, 38% were somewhat to extremely concerned about communicating with the trial team and 43% were somewhat to extremely concerned about a lack of connection or relationship with the study team.

**Table 3:** Preferences related to participating in a decentralized trial

Question and Response Options	Responses
	Average order of importance*
One person as your go-to contact, for example a research coordinator	1.94
Access to a 24-hour help line that has someone responding in real-time to all enquiries via call or email or live chat	2.85
Instructions on what to do if there is an emergency in ‘off hours’ and having the information you need to handle it	3.13
Ability to send a text message for support	3.28
An information hub (e.g., on a website or a physical pamphlet/flyer with information on it)	3.81
**If you participated in a decentralized clinical trial how concerned would you be about the following**:	
**How to handle adverse events.**	
Extremely concerned	33 (13.4%)
Moderately concerned	58 (23.6%)
Somewhat concerned	61 (24.8%)
Slightly concerned	68 (27.6%)
Not at all concerned	26 (10.6%)
**How to handle any potential complications.**	
Extremely concerned	40 (16.3%)
Moderately concerned	55 (22.4%)
Somewhat concerned	68 (27.6%)
Slightly concerned	62 (25.2%)
Not at all concerned	21 (8.5%)
**The overall quality of the research being different than in-person because data, samples, etc. were collected at locations other than the study site.**	
Extremely concerned	11 (4.4%)
Moderately concerned	32 (12.8%)
Somewhat concerned	55 (22.0%)
Slightly concerned	70 (28.0%)
Not at all concerned	82 (32.8%)
**Technology access and use.**	
Extremely concerned	10 (4.0%)
Moderately concerned	23 (9.2%)
Somewhat concerned	37 (14.9%)
Slightly concerned	60 (24.1%)
Not at all concerned	119 (47.8%)
**Communication with the trial team.**	
Extremely concerned	19 (7.6%)
Moderately concerned	35 (14.5%)
Somewhat concerned	40 (16.1%)
Slightly concerned	71 (28.5%)
Not at all concerned	83 (33.3%)
**Lack of connection or relationship with the trial team.**	
Extremely concerned	19 (7.7%)
Moderately concerned	43 (17.3%)
Somewhat concerned	44 (17.7%)
Slightly concerned	77 (31.0%)
Not at all concerned	65 (26.2%)
**If you participated in a decentralized clinical trial, what do you think might be some potential benefits to you? Please mark all that apply.**	
Easier to participate overall	217 (87.5%)
Flexibility related to participation	186 (75.0%)
Less time investment	138 (55.6%)
Costs me less	138 (55.6%)

*A low average indicates increased order of importance. For example, an average score of 1 would represent the greatest possible order of importance whereas an average score of 5 would represent the least possible order of importance

Lastly, when asked to identify some potential benefits of participating in a decentralized trial, 88% thought that it would be easier to participate overall followed by: flexibility (75%), less time investment (56%) and cost (56%).

### Informed Consent

Table 4 presents respondents’ preferences to questions related to providing informed consent. Of note: 85% of the participants indicated a moderate to great deal of preference to providing informed consent at a satellite site closer to home rather than at the main study site, 80% indicated a moderate to great deal of preference to providing informed consent via video conference or some other technology-based application, and 61% indicated a moderate to great deal of preference to providing informed consent on the phone.

**Table 4:** Informed consent preferences

Question and Response Options	Responses	Question and Response Options	Responses
***How strongly do you prefer/not prefer the following ways to provide informed consent to participate in a clinical trial?***		***If you provided informed consent to participate in a clinical trial on the phone or by videoconference (e.g., using an application such as Zoom), rate your level of concern about the following statements:***	
**In person at the study site.**		**How concerned would you be about not feeling as comfortable asking questions as you would be if you were in person?**	
Prefer a great deal	25 (14.1%)	Extremely concerned	1 (0.6%)
Prefer a lot	22 (12.4%)	Moderately concerned	11 (6.4%)
Prefer a moderate amount	43 (24.3%)	Somewhat concerned	12 (7.0%)
Prefer slightly	23 (13.0%)	Slightly concerned	26 (15.1%)
Do not prefer	64 (36.2)	Not at all concerned	122 (70.9%)
**At a ‘satellite’ site that is closer to your home than the main study site.**		**How concerned would you be that the quality of the interaction or communications might not be as good as if you were in person?**	
Prefer a great deal	28 (16.0%)	Extremely concerned	7 (4.0%)
Prefer a lot	58 (34.3%)	Moderately concerned	22 (12.7%)
Prefer a moderate amount	60 (34.3%)	Somewhat concerned	24 (13.9%)
Prefer slightly	36 (20.6%)	Slightly concerned	45 (26.0%)
Do not prefer	16 (9.1%)	Not at all concerned	75 (43.4%)
**On the phone.**		**How concerned would you be about the quality of the relationship you build with the clinical trial team?**	
Prefer a great deal	20 (11.4%)	Extremely concerned	10 (5.8%)
Prefer a lot	39 (22.3%)	Moderately concerned	23 (13.3%)
Prefer a moderate amount	48 (27.4%)	Somewhat concerned	28 (16.2%)
Prefer slightly	27(15.4%)	Slightly concerned	43 (24.9%)
Do not prefer	41 (23.4%)	Not at all concerned	69 (39.9%)
**Via videoconference or some other technology-based application.**		**How concerned would you be about not being able to talk to the person on the clinical trial team you would like to? (e.g., Investigator, clinical trial nurse, etc.)**	
Prefer a great deal	53 (30.3%)	Extremely concerned	13 (7.5%)
Prefer a lot	57 (32.6%)	Moderately concerned	38 (22.0%)
Prefer a moderate amount	29 (16.6%)	Somewhat concerned	28 (16.2%)
Prefer slightly	22 (12.6%)	Slightly concerned	54 (31.2%)
Do not prefer	14 (8.0%)	Not at all concerned	40 (23.1%)
**Via secure email (e.g., digital signature through an app like DocuSign)**		**How concerned would you be about your privacy?**	
Prefer a great deal	43 (24.6%)	Extremely concerned	12 (6.9%)
Prefer a lot	44 (25.1%)	Moderately concerned	22 (12.7%)
Prefer a moderate amount	32 (18.3%)	Somewhat concerned	15 (8.7%)
Prefer slightly	26 (14.9%)	Slightly concerned	36 (20.8%)
Do not prefer	30 (17.1%)	Not at all concerned	88 (50.9%)
		**How concerned would you be about technology problems or difficulty using a technology?**	
		Extremely concerned	10 (5.8%)
		Moderately concerned	15 (8.7%)
		Somewhat concerned	18 (10.4%)
		Slightly concerned	52 (30.1%)
		Not at all concerned	78 (45.1%)
		**How concerned would you be about language barriers and potentially not having a caregiver present with you, not having interpreters available, etc.?**	
		Extremely concerned	2 (1.2%)
		Moderately concerned	10 (5.8%)
		Somewhat concerned	4 (2.3%)
		Slightly concerned	16 (9.2%)
		Not at all concerned	141 (81.5%)

When asked further questions related to providing informed consent via phone or teleconference, most respondents indicated an overall low level of concern. However, when asked about how concerned they would be about privacy, 50% of respondents indicated they were at least slightly concerned.

### Travel

Table 5 presents responses to questions related to travel. Slightly more than one-third of respondents (35%) were willing to travel more than one hour to participate in a clinical trial and 39% preferred in-person interaction with staff even if it required more travel. When asked to rank factors in order of importance related to how long they would be willing to travel, the most important factor was what the trial was for, followed by quality of life, how many times travel was required, travel related costs, time, and how much they or others would be inconvenienced. Respondents had a clear preference for routine and simple tests to be taken at a private lab or close to home (72%).

**Table 5:** Preferences related to travel and visits

Question and Response Options	Responses
**How long would you be willing to travel to participate in a clinical trial?**	
Less than one hour	113 (64.6%)
More than one hour	62 (35.4%)
**Please rank in order of importance what would influence your preference for how long you would be willing to travel to participate in a clinical trial.**	
	Average order of importance*
What the trial is for	2.86
What my quality of life is	3.37
How many times I might be required to travel throughout the trial	3.42
If the cost of travel and accommodations or the cost of my time is covered	3.42
Time	3.52
How much this might inconvenience myself and/or a caregiver who would accompany me to visits	4.40
**Would you prefer in-person interaction with site staff during visits even if it required more travel and potential inconvenience?**	
Yes	23 (13.1%)
No	69 (39.2%)
Maybe	84 (47.7%)
**Please indicate your reasons for responding yes to the question “Would you prefer in-person interaction with site staff during visits even if it required more travel and potential inconvenience?”**	
	Checked
Concerns about the interaction or communications might not be as good as if you were in person	17 (73.9%)
Concerns about the quality of the relationship you build with the clinical trial team	16 (69.6%)
Concerns about not being able to talk to the person on the clinical trial team when you want to	13 (56.5%)
Concerns about not feeling as comfortable asking questions as you would be in person	9 (39.1%)
Concerns about using technology	5 (21.7%)
Concerns about privacy	5 (21.7%)
Concerns about safety	3 (13.0%)
Concerns about language barriers	2 (8.7%)
**If required for the clinical trial, where would you prefer routine and simple tests be done or samples taken?**	
At a private lab close to home	127 (72.6%)
At your home	60 (34.3%)
At a study site	25 (14.3%)
No preference	20 (11.4%)

*A low average indicates increased order of importance. For example, an average score of 1 would represent the greatest possible order of importance whereas an average score of 5 would represent the least possible order of importance

### Data Collection, Keeping a Patient Diary, home Delivery of Study Materials, and Accessibility

Complete results of the questions related to data collection, keeping a patient diary, home delivery of study materials, and accessibility are in Supplementary Materials.

When asked questions about concerns regarding entering study data for the trial from home, most respondents (59%) were not at all concerned with providing their information via technology. However, a substantial proportion of respondents were at least somewhat concerned with the possibility of a third party accessing their records (43%) or the overall privacy of the shared data (40%).

Respondents showed a clear preference for keeping a patient diary via typed or voice technology on an app, their home computer or a device provided to them by the study team. Those who preferred a technology-based option indicated that the provision of clear instructions would be most helpful (90%), followed by access to IT support (72%), knowing their account would be deleted at the end of the study (55%), not needing a password or log in requirements (26%), and having wifi or internet supplied to them by the study team (17%).

We also asked the respondents to rank a selected list of concerns related to having an investigational product delivered to their home. The top concerns were related to having clear instructions given to them by someone visiting their home or via technology, followed by being offered a timeframe for delivery and issues regarding security and having others in their home.

Lastly, most respondents thought that decentralized trials would improve accessibility for all people in Canada (91%) and potentially increase the diversity of clinical trial participants (85%).

There were some opportunities for respondents to provide open-ended text responses and at the end of the survey, a catch all open-ended request for comments about anything else respondents wished to share. These responses were categorized and tallied as follows: access concerns or thoughts (29 responses), financial concerns (2 responses), technological concerns (4 responses) and travel concerns (10 responses).

## Discussion

We present here what we believe are the first survey results of people in Canada on their perceptions related to participating in decentralized or hybrid clinical trials. The idea for the survey emerged from discussions between CTO’s College of Lived Experience and members of the Ontario clinical trials community early in the pandemic. Individuals conducting clinical trials were interested in understanding participant and potential participant views on decentralized and hybrid clinical trials, and we saw value in extending that beyond our limited number of College members. Knowing what potential participants think and feel about these types of trials in the form of survey evidence may influence those doing trials to operationalize these perspectives and may potentially facilitate the long-term uptake of decentralized and hybrid trials.

*“It’s about time we bring trials to people. As someone with many health issues, I already have a lot on my plate and more needs to be done to make participation in clinical trials simple and easy to access.”* - Survey respondent

Our online survey was promoted via online mechanisms and through a number of health charities and patient organizations with whom CTO has collaborative relationships. The demographics of the respondents to our survey (Table 1) indicate that the respondents were primarily middle-aged, Caucasian women with a high level of education and income, and is supported by the literature relating to this recruitment approach [9]. However the high response rate from women about clinical trials is interesting given that they are among a population that has historically been excluded from trials for a variety of reasons [25]. Furthermore, patient organizations’ and health charities’ audiences and communities may be more experienced with respect to clinical trial participation, and may be more knowledgeable and aware of clinical trials than members of the general public. The number of survey respondents who indicated they have “participated in a clinical trial” and “have cared for someone who has participated” is a much higher number than reported in a survey we conducted of the general public (in Ontario and British Columbia, in 2015) where those responses were 11% (vs. 40% in the current survey) and 7% (vs. 29%), respectively [26]. With respect to knowledge levels of clinical trials, respondents in this survey felt much more informed (felt partially informed, informed and very informed) about clinical trials, compared to our 2015 survey of the general public where 57% of respondents felt somewhat or very informed vs. 88% in the current survey (note that the response options were not identical, so we have taken liberties with a direct comparison) [26].

Our main takeaway from the results of this survey is that potential participants are seeking options related to decentralized and hybrid clinical trials. Nearly 80% of respondents indicated that they want a range of options for in-person, at home and virtual site visits. This finding is similar to findings in the annual CISCRP Perceptions and Insights Study Report (2021), although our survey respondents’ breakdown of type of options and preferences varied from those in the CISCRP report and those of rare disease patients [13, 27]. Comparably, survey results of patients with cancer or cancer survivors about clinical trials showed a willingness to adopt technology and other decentralized tools (60–85%) when this would result in less travel and less time to participate [28]. Our survey respondents were nearly evenly split on being concerned or not at all concerned about using and accessing technology, which is similar to findings with rare disease patients [13].

*“The pandemic forced us to innovate in order to adapt to restrictions on in-person visits. I worry...those gains will be lost over time as people slip back into the status quo.”*- Survey respondent

A member of CTO’s College coined the phrase ‘nuisance factors’ to describe items that represent potential burdens or barriers to participation. For example, one ‘nuisance factor’ is the time needed to travel to a site visit for a clinical trial. Nearly 65% of our survey respondents indicated that they would want this site travel time to be less than one hour. In one survey of oncology patients, 13% of respondents indicated they would be agreeable with driving between 45–90 minutes to a site [12], whereas in another survey, 30% of respondents were willing to travel up to 90 minutes or more to be in a trial and 77% would join if the trial was comparable in distance and frequency to their regular care [28]. A survey of rare disease patients indicated that 60% of them appreciated less travel to sites [13].

*“This [decentralized or hybrid trials] would be a great option for people with restricted mobility or for those that have limited transportation options.”*– Survey respondent

Our survey results indicate that people have concerns about decentralized or hybrid trials in regards to communications with the trial team (67% were concerned), their overall relationship with the trial team (74% were concerned), and quality of the research (67% were concerned). These communications and relationships findings contrast with the results of a perspectives study of individuals in the UK and Denmark, which found that 24% of respondents were concerned about missing in-person site visits [14]. Our survey respondents indicated that they may have concerns about handling adverse events and potential complications, which is also aligned with other survey results [12, 13]. CTO’s College members and survey respondents expressed concerns about the quality of research, which was also of concern in the oncology community (expressed as ‘data integrity’ or ‘unreliable results’) [12, 14]. On maintaining relationships and communication, one idea from the literature is to have the trial team communicate on a regular basis (e.g., monthly) with the participant, so as to keep the relationship ties, for motivational purposes, and to provide access to experts [14].

The literature cites potential ethical concerns about informed consent being performed virtually or through other technologies, which we probed in our survey [7]. The questions in our survey appear unique compared to the literature on preferences related to decentralized or hybrid trials. The responses here continue to follow a theme related to a preference for providing options, with most preferring videoconference or in-person at a satellite site closer to home, while the fewest preferring in-person at the study site. Options including over the phone and via a secure application were not preferred, perhaps due to not being able to see the person during the process or due to perceived difficulties with secure applications. With respect to providing consent in a non-face-to-face manner, there was a high degree of being “not at all concerned” about asking questions, the quality of the interaction or speaking to a specific person on the team, the quality of the relationship with the trial team, privacy, technology, and language barriers. The response in particular to language barriers likely represents a bias in our sample of English-speakers (though we did not ask about languages spoken). These responses may generally be reflective of how society has become accustomed to virtual methods of interacting and communicating throughout the pandemic.

*“Sites need better support and infrastructure to go to remote based studies and REB [Research Ethics Board] support as well to allow consent process flexibility.”* - Survey respondent

The majority of participants in our survey felt that decentralized trials could offer personal benefits related to ease of participation, flexible participation, less time involvement, and less cost. These may all relate to an increased quality of life as cited by the survey of oncology patients which may also lead to increase retention [12]. CTO’s College members also posited that decentralized and hybrid trials may lead to increased recruitment, and less ‘burnout’ for participants, especially in rare disease communities where the clinical trial pipeline is currently robust (e.g., Huntington’s disease). Our survey respondents agreed overwhelmingly that decentralized trials would improve accessibility for all people in Canada and potentially increase the diversity of clinical trial participants, cited as potential benefits by a number of others [9, 10].

*“Access to clinical trials especially for those who live outside a major urban Center [sic] is extremely important.”*- Survey respondent

Our survey results support the importance of ongoing efforts by regulators and other organizations to ensure that decentralized and hybrid clinical trials are feasible and safely operationalized to address the preferences and concerns of potential participants. Remote data acquisition in clinical trials and safe oversight of decentralized procedures are among topics being actively address by regulators [5, 29] and clinical trial organizations [30–32]. When exploring key considerations to operationalize decentralized and hybrid clinical trials, the clinical trial community in Ontario expressed an importance to understanding what potential participants in Canada would prefer. These survey results provide evidence to inform regulators, sponsors, REBs, and clinical trial teams in these efforts. The survey results indicate that respondents would prefer options for participation, and which may point to the preference for hybrid approaches rather than the all-or-nothing approach of the decentralized clinical trial definition [5].

Our authorship team has patient and public engagement learnings to share with the wider community, having worked with CTO’s College of Lived Experience from the idea phase of this project to this publication. First, College members feel that bringing their lived experiences in to designing the survey led to the relevance of the questions. While we allowed for open-ended text responses in the survey and at the end of the survey to comment on any items that respondents felt were missing, there were no additional topics identified by respondents. Instead, respondents tended to provide additional context to their responses in these open-ended fields. College members feel a sense of reward in that these survey results may influence how trials are done in the future, and that may mean lessening the burden for other participants through options that were not provided in the past to them when they participated in trials. College members feel that they were included throughout this project as team members who actively contributed to the project, were listened to, and can see the impact of their inputs in this work. It is noteworthy that resources went in to the work to engage the College in this project in the form of hosting numerous virtual meetings (group and one on one) over the period of the project, including in the form of a dedicated specialist (DPR) who worked with the College, an epidemiologist (JQ) who was brought on to help with the survey and its analysis, and an honorarium for College members who wished to receive it. It is also likely that the project took longer than it would have if the CTO team would not have collaborated with the College, though we are all too aware that the result would not have been the same.

In terms of study limitations, we have covered the demographic of the majority of survey respondents which may provide a bias in results. Few respondents represented rural perspectives (less than 9%), leaving the survey responses being mostly representative of those in urban settings. This was an online survey that was promoted mainly through CTO or patient organizations and health charities with whom CTO works. In order to be aware of the survey, respondents would likely have had to be knowledgeable about CTO or a patient organization or health charity. We have also covered above how the respondents to our survey may potentially have more experience as clinical trial participants in or are more knowledgeable in general about clinical trials. While our study was approved by an REB, we found that some local REBs would not allow sharing of the survey if their local board had not reviewed and approved the survey. An internet connection was required to be able to participate in this online survey. And lastly, due to the number of respondents to our survey, we were not able to find statistically significant differences to responses based on gender, age, health status, geography, ethnicity or prior clinical trial participation.

## Conclusion

We present what we believe to be the first findings related to perceptions and perspectives on decentralized and hybrid clinical trials based on a survey of people in Canada. Our survey and this publication are the result of our work with a group of individuals with lived experiences and who informed all project aspects. Our survey highlights that people in Canada wish to see a range of options for participating in clinical trials. This wish should be considered by those conducting trials and those creating policies and regulations related to clinical trials in Canada (and beyond).

### Electronic Supplementary Material

Below is the link to the electronic supplementary material.

Supplementary Material 1 - CHERRIES Checklist

Supplementary Material 2 - GRIPP2 Checklist

Supplementary Material 3 - Full Survey

Supplementary Material 4 - Results of questions related to data collection, keeping a patient diary, home delivery of study materials, and accessibility
