# Supplementary material for: Patient and Public Perceptions in Canada About Decentralized and Hybrid Clinical Trials: “It’s About Time we Bring Trials to People”
Source: Ther Innov Regul Sci. 2024 Jun 21;58(5):965–77. doi: 10.1007/s43441-024-00665-y (PMC11335844; doi:10.1007/s43441-024-00665-y)
Supplement: Supplementary file 2 — Supplementary Material 2 - GRIPP2 Checklist [file 43441_2024_665_MOESM2_ESM.docx]

**Guidance for Reporting Involvement of Patients and the Public (GRIPP2) Checklist**

| **Section and Topic** | **Item** | **Page number** |
| --- | --- | --- |
| 1. Aim | Report the aim of PPI in the study | 4 |
| 2. Methods | Provide a clear description of the methods used for PPI in the study | 6-7 |
| 3. Study results | Outcomes – Report the results of PPI in the study, including both positive and negative outcomes | 18-19 |
| 4. Discussion and conclusions | Outcomes – Comment on the extent to which PPI influenced the study overall. Describe positive and negative effects. | 18-19 |
| 5. Reflections/critical perspective | Comment critically on the study, reflecting on things that went well and those that did not, so others can learn from this experience. | 18-19 |
