# Supplementary material for: Patient and Public Perceptions in Canada About Decentralized and Hybrid Clinical Trials: “It’s About Time we Bring Trials to People”
Source: Ther Innov Regul Sci. 2024 Jun 21;58(5):965–77. doi: 10.1007/s43441-024-00665-y (PMC11335844; doi:10.1007/s43441-024-00665-y)
Supplement: Supplementary file 3 — Supplementary Material 3 - Full Survey [file 43441_2024_665_MOESM3_ESM.docx]

**Supplementary Material 1**

**Survey of the attitudes and preferences of Canadians towards participating in a decentralized clinical trial**

Start of Block: Informed Consent

Q1
Welcome   

We are inviting you to participate in a research project entitled “Survey of the attitudes and preferences of Canadians towards participating in a decentralized clinical trial”. The survey is being conducted by Dr. Dawn Richards and Ms. Susan Marlin of Clinical Trials Ontario (CTO) and Drs. John Queenan and Bradley Stoner of Queen’s University.

We are interested in understanding your thoughts and opinions related to participating in a clinical trial. For this study, you will be presented with information relevant to participating in clinical trials in Canada. Then, you will be asked to answer some questions. Your responses will be kept completely confidential.  

 The questionnaire is split into two sections.  The first section should only take about ten minutes to complete.  The second section is optional and should take a further ten minutes to complete.
 
Background and Purpose  
 
Clinical trials are a type of research that involve people and test health-related “interventions” to see how they work. There are many types of interventions tested in clinical trials. While we often think about clinical trials being done to test drugs, they also test devices, genetic therapies, natural health products, psychotherapies, lifestyle and preventive care interventions, and many other things. The interventions studied in clinical trials can be very experimental, with little to no evidence about how they might work in people (e.g. new drugs). Or they may already be commonly used by people but more evidence is needed about how they work. 
 
Clinical trials are carefully designed and planned to make sure that risks to the people participating are minimized to the extent possible, and that the questions asked about the intervention(s) can be answered when the trial is finished. 
 
**Traditional clinical trials** usually take place at trial sites where an Investigator and study staff do procedures related to the clinical trial. Using this approach, participants go to the study team at a study site to take part in a clinical trial.
 

 **Decentralized clinical trials** (abbreviated as DCTs and also sometimes called remote or virtual clinical trials) are clinical trials that occur with participants that are not required to come to a study site. Participants may use videoconferencing or other technology to talk to study staff, be visited at home by study staff, use internet-based tools for collecting data and reporting, and use mobile technology (e.g., a wearable technology).

 The COVID-19 pandemic has led to some clinical trials being done differently from how they were originally planned. Health Canada, Canada’s regulatory agency that oversees clinical trials done in Canada, has allowed these changes to happen during the pandemic and is also looking at how some of these temporary changes might be allowed to continue in the future. 

This survey is being hosted by Clinical Trials Ontario (also called CTO), a not for profit organization dedicated to making Ontario a better place to do clinical trials. CTO works with the clinical trials community in Ontario and beyond and that includes working with patients and the public. We co-developed this survey with patients, health charities, patient organizations, and individuals who are involved in facilitating and doing clinical trials.     
 
Privacy and Survey Participation

Please be assured that your participation is entirely voluntary, and you may decline to participate and refuse to answer any or all of the questions without penalty. However, your data cannot be withdrawn after the submission of the survey.  Should you decide to withdraw prior to submission you may do so simply by exiting your browser. We will not be collecting any directly identifiable information from you.  All of your answers will be anonymous and kept confidential; we will not identify you in any reports or publications. There are no direct benefits to you nor will there be any compensation if you choose to answer the survey.  There are no known risks to participating in the survey.  The study data will be stored on Queen’s University privacy approved servers hosted by Qualtrics.  We will keep your data securely for at least five years per Queen’s University Policy, after which the study data will be securely destroyed.  Only Dr. John Queenan and Ms. Susan Marlin will have access to the survey data.  

 If you have any questions or concerns about the project, feel free to contact John Queenan at 1-416-673-6684. 

This study has been reviewed for ethical compliance by the Queen's University Health Sciences and Affiliated Teaching Hospitals Research Ethics Board (HSREB). The HSREB may require access to your study-related records to monitor the ethical conduct of the research. If you have any ethics concerns, please contact the Queen’s University Health Sciences and Affiliated Teaching Hospitals Research Ethics Board (HSREB) at 1-844-535-2988 (Toll free in North America) or [hsreb@queensu.ca](mailto:hsreb@queensu.ca).

A copy of this letter of information and consent can be downloaded by clicking on this link: Letter of information and consent

By clicking below, I am verifying that: I have read the above Letter of Information, have had all my questions answered, I have not waived any legal rights in the event of research-related harm and that I consent to participate in this research study.

I consent, begin the study (1)

I do not consent, I do not wish to participate (2)

End of Block: Informed Consent

Start of Block: Preferences and Perceptions related to clinical trial participation

Q24 **First, we will ask some questions about your preferences and perceptions related to clinical trial participation. Respond to these questions based on your personal situation rather than thinking about a specific type of trial. At the end of the survey you will be given the chance to share additional thoughts.**

Q25 When thinking about the different ways you could participate in a clinical trial, how important is it to you to be provided with options for where to have your study visits (for example, study visits at a clinical trial site, virtual or in-person visits at home, etc.)?

- Not at all important (1)
- Slightly important (2)
- Moderately important (3)
- Very important (4)
- Extremely important (5)

| Page Break |  |
| --- | --- |

Q27 **How strongly do you agree/disagree with the following statements regarding your preference for being willing to participate in each of the following types of clinical trials?**

Q28 I would prefer to have all my study visits at the study clinic and seeing the study doctor and staff in-person at the clinic only.

- Strongly disagree (1)
- Somewhat disagree (2)
- Neither agree nor disagree (3)
- Somewhat agree (4)
- Strongly agree (5)

| Page Break |  |
| --- | --- |

Q29 I would prefer to have some study visits at my home and some visits at the study clinic, and seeing the study doctor sometimes via video conference from home or in-person at the clinic.

- Strongly disagree (1)
- Somewhat disagree (2)
- Neither agree nor disagree (3)
- Somewhat agree (4)
- Strongly agree (5)

| Page Break |  |
| --- | --- |

Q30 I would prefer having a nurse or study team member come to my home for all of my study visits and seeing the study doctors via video conference from home.

- Strongly disagree (1)
- Somewhat disagree (2)
- Neither agree nor disagree (3)
- Somewhat agree (4)
- Strongly agree (5)

Q31 I would prefer to collect all of my health data at home using technology and only talking to the study team via video conference from home.

- Strongly disagree (1)
- Somewhat disagree (2)
- Neither agree nor disagree (3)
- Somewhat agree (4)
- Strongly agree (5)

End of Block: Preferences and Perceptions related to clinical trial participation

Start of Block: Questions about Participating in a Decentralized Trial

Q72 If you participated in a decentralized clinical trial, what types of communications with the clinical trials team would be most helpful to you? Please rank in order of importance.  Use your mouse to drag your selections and place them in your preferred order.  If you are using a phone or tablet use your finger.

______ One person as your go-to contact, for example a research coordinator (1)

______ Access to a 24 hour help line that has someone responding in real-time to all enquiries via call or email or live chat (2)

______ Ability to send a text message for support (3)

______ An information hub (e.g. on a website or a physical pamphlet/flyer with information on it) (4)

______ Instructions on what to do if there is an emergency in ‘off hours’ and having the information you need to handle it (5)

Q73 Please let us know if anything else may be helpful regarding communications.

________________________________________________________________

| Page Break |  |
| --- | --- |

Q74 If you participated in a decentralized clinical trial, what do you think might be some potential benefits to you? Please mark all that apply.

- Less time investment (1)
- Easier to participate overall (2)
- Costs me less (3)
- Flexibility related to participation (4)

| Page Break |  |
| --- | --- |

Q75 If you participated in a decentralized clinical trial how concerned would you be about the following:

Q76 How to handle adverse events.

- Not at all concerned (1)
- Slightly concerned (2)
- Somewhat concerned (3)
- Moderately concerned (4)
- Extremely concerned (5)

Q77 How to handle any potential complications.

- Not at all concerned (1)
- Slightly concerned (2)
- Somewhat concerned (3)
- Moderately concerned (4)
- Extremely concerned (5)

| Page Break |  |
| --- | --- |

Q78 The overall quality of the research being different than in-person because data, samples, etc. were collected at locations other than the study site.

- Not at all concerned (1)
- Slightly concerned (2)
- Somewhat concerned (3)
- Moderately concerned (4)
- Extremely concerned (5)

Q79 Technology access and use.

- Not at all concerned (1)
- Slightly concerned (2)
- Somewhat concerned (3)
- Moderately concerned (4)
- Extremely concerned (5)

Q80 Communication with the trial team.

- Not at all concerned (1)
- Slightly concerned (2)
- Somewhat concerned (3)
- Moderately concerned (4)
- Extremely concerned (5)

| Page Break |  |
| --- | --- |

Q81 Lack of connection or relationship with the trial team.

- Not at all concerned (1)
- Slightly concerned (2)
- Somewhat concerned (3)
- Moderately concerned (4)
- Extremely concerned (5)

End of Block: Questions about Participating in a Decentralized Trial

Start of Block: Health Questions

Q81 **We would like to ask you a few personal questions including ones about your health, education and income. Please be assured that all information collected will be held in the strictest of confidence and you will not be identified in any reports created from the information we are gathering. If you do not want to answer any of these questions please feel free to simply skip to the next question.**

| Page Break |  |
| --- | --- |

Q18 How would you consider your health?

- Very Poor (1)
- Poor (2)
- Fair (3)
- Good (4)
- Excellent (5)

Q19 Do you live with a chronic health condition, and if so, how many? A chronic condition is defined as a condition that lasts for 1 year or more and needs ongoing medical care or limit activities of daily living or both.  Some examples of common chronic conditions are diabetes, arthritis, heart disease or depression.  Some cancers such as those of the breast, prostate and colon may also be considered chronic conditions.

- None (1)
- One (2)
- Two (3)
- Three or more (4)

| Page Break |  |
| --- | --- |

Q20 Do you have a life-threatening illness now or have you in the past?

- Yes (1)
- No (2)
- Unsure (3)

Q21 Have you ever participated in a clinical trial?

- Yes (1)
- No (2)
- Unsure (3)

| Page Break |  |
| --- | --- |

Q22 Has someone you care for ever participated in a clinical trial (e.g. a spouse, child, parent, etc.)?

- Yes (1)
- No (2)
- Unsure (3)

Q23 Overall, how would you rate your knowledge of clinical trials?

- Not at all informed (1)
- Somewhat informed (2)
- Partially informed (3)
- Informed (4)
- Very Informed (5)

End of Block: Health Questions

Start of Block: Demographics Questions

Q6 **Demographics Questions: We would like to know a bit more about you. Please remember that if you do not want to answer any of these questions please feel free to simply skip to the next question.**

| Page Break |  |
| --- | --- |

Q7 Please indicate the first letter and following number of your postal code.  For example, if your postal code is X9X 9X9 please input "X"  as the letter and 9 as the number.  The first two characters of the postal code will allow us to know in which province you live and whether or not you live in an urban or rural area.  We will not be using this information to attempt to identify you in any way.

- First Letter (4) ________________________________________________
- Following Number (5) ________________________________________________

Q8 How old are you in years?  If you prefer not to answer please leave blank.

________________________________________________________________

Q10 Please select the option that best describes you.

- Woman (1)
- Man (2)
- Gender fluid, non-binary, and/or Two-Spirit (3)
- Prefer not to answer (4)

| Page Break |  |
| --- | --- |

Q11  Please indicate the highest level of education you have achieved:

- High school or less (1)
- Some college or trade school or university (2)
- Completed college or trade school certificate or diploma (3)
- University Undergraduate (4)
- University Graduate Level/Postgraduate/Professional (MA, MSc., PhD., Law School, Medical School) (5)
- Prefer not to answer (6)

| Page Break |  |
| --- | --- |

Q13 What best describes your ethnic background? (check all that apply)

- White/Caucasian (1)
- Chinese (2)
- South Asian (3)
- Black (4)
- Filipino (5)
- Latin American (6)
- Southeast Asian (7)
- Arab (8)
- West Asian (9)
- Japanese (10)
- Korean (11)
- Other (12) ________________________________________________

| Page Break |  |
| --- | --- |

Q15 Which best describes your current daily life? (check all that apply).

- working at a full-time job (1)
- working at a part-time job (2)
- self-employed (including working in a gig-economy job) (3)
- looking for work, in between jobs (4)
- on maternity/paternity leave (5)
- on long-term disability (6)
- homemaking/caregiving (7)
- volunteering (8)
- going to school (9)
- retired (10)
- Prefer not to answer (11)
- Other (12) ________________________________________________

| Page Break |  |
| --- | --- |

Q17 What is your pre-tax annual household income?

- Less than $15,000 (1)
- $15,000-$29,999 (2)
- $30,000-$49,999 (3)
- $50,000-$74,999 (4)
- $75,000 – $99,999 (5)
- More than $100,000 (6)
- Prefer not to answer (7)

End of Block: Demographics Questions

Start of Block: Closing Questions

Q84 Are there other aspects of decentralized clinical trials that might concern you that we did not ask about?

________________________________________________________________

Q85 Is there anything else you wish to comment on that we did not ask about or that you would like us to know about?

________________________________________________________________

| Page Break |  |
| --- | --- |

Q82 This is the end of the first section of the questionnaire. Would you like to continue and answer some more specific questions regarding your clinical trial preferences?

- No (1)
- Yes (2)

End of Block: Closing Questions

Start of Block: Questions about Informed Consent

Q32 **A key part of the informed consent processes for clinical trials is a document called the ‘informed consent form’ or ‘consent form’. This document should provide all relevant information about the clinical trial and be written in a way that is understandable to you. You should be given enough time to read it and ask any questions you may have. If you agree to participate, you may be asked to sign the consent form and should be provided with a copy of the form for your own keeping.

 How strongly do you prefer/not prefer the following ways to provide informed consent to participate in a clinical trial?**

Q33 In person at the study site.

- Do not prefer (1)
- Prefer slightly (2)
- Prefer a moderate amount (3)
- Prefer a lot (4)
- Prefer a great deal (5)

| Page Break |  |
| --- | --- |

Q34 At a ‘satellite’ site that is closer to your home than the main study site

- Do not prefer (1)
- Prefer slightly (2)
- Prefer a moderate amount (3)
- Prefer a lot (4)
- Prefer a great deal (5)

Q35 On the phone.

- Do not prefer (1)
- Prefer slightly (2)
- Prefer a moderate amount (7)
- Prefer a lot (8)
- Prefer a great deal (9)

| Page Break |  |
| --- | --- |

Q36 Via videoconference or some other technology-based application.

- Do not prefer (1)
- Prefer slightly (2)
- Prefer a moderate amount (3)
- Prefer a lot (4)
- Prefer a great deal (5)

| Page Break |  |
| --- | --- |

Q37 Via secure email (e.g. digital signature through an app like DocuSign)

- Do not prefer (1)
- Prefer slightly (2)
- Prefer a moderate amount (3)
- Prefer a lot (4)
- Prefer a great deal (5)

Q38 Other (please explain)

________________________________________________________________

End of Block: Questions about Informed Consent

Start of Block: Questions about Providing consent by phone or videoconference

Q40 **If you provided informed consent to participate in a clinical trial on the phone or by videoconference (e.g. using an application such as Zoom), rate your level of concern about the following statements:**

Q41 How concerned would you be about not feeling as comfortable asking questions as you would be if you were in person?

- Not at all concerned (1)
- Slightly concerned (2)
- Somewhat concerned (3)
- Moderately concerned (4)
- Extremely concerned (5)

| Page Break |  |
| --- | --- |

Q42 How concerned would you be that the quality of the interaction or communications might not be as good as if you were in person?

- Not at all concerned (1)
- Slightly concerned (2)
- Somewhat concerned (3)
- Moderately concerned (4)
- Extremely concerned (5)

Q43 How concerned would you be about the quality of the relationship you build with the clinical trial team?

- Not at all concerned (1)
- Slightly concerned (2)
- Somewhat concerned (3)
- Moderately concerned (4)
- Extremely concerned (5)

| Page Break |  |
| --- | --- |

Q45 How concerned would you be about not being able to talk to the person on the clinical trial team you would like to? (e.g., Investigator, clinical trial nurse, etc.)

- Not at all concerned (1)
- Slightly concerned (2)
- Somewhat concerned (3)
- Moderately concerned (4)
- Extremely concerned (5)

Q46 How concerned would you be about your privacy?

- Not at all concerned (1)
- Slightly concerned (2)
- Somewhat concerned (3)
- Moderately concerned (4)
- Extremely concerned (5)

| Page Break |  |
| --- | --- |

Q47 How concerned would you be about technology problems or difficulty using a technology?

- Not at all concerned (1)
- Slightly concerned (2)
- Somewhat concerned (3)
- Moderately concerned (4)
- Extremely concerned (5)

Q44 How concerned would you be about language barriers and potentially not having a caregiver present with you, not having interpreters available, etc?

- Not at all concerned (1)
- Slightly concerned (2)
- Somewhat concerned (3)
- Moderately concerned (4)
- Extremely concerned (5)

Q45 Please let us know if you have any other concerns that we have not directly addressed above.

________________________________________________________________

End of Block: Questions about Providing consent by phone or videoconference

Start of Block: Questions about Travel

Q46 How long would you be willing to travel to participate in a clinical trial?

- Less than one hour (1)
- More than one hour (2)

| Page Break |  |
| --- | --- |

Q47 Please rank in order of importance what would influence your preference for how long you would be willing to travel to participate in a clinical trial.

______ Time (1)

______ If the cost of travel and accommodations or the cost for my time is covered (2)

______ What the trial is for (3)

______ What my quality of life is (4)

______ How much this might inconvenience myself and/or a caregiver who would accompany me to visits (5)

______ How many times I might be required to travel throughout the trial (6)

| Page Break |  |
| --- | --- |

Q48 Please use the space below to let us know if there is anything else that would influence your preference for how long you would be willing to travel to participate in a clinical trial.

________________________________________________________________

Q49 Would you prefer in-person interaction with site staff during visits even if it required more travel and potential inconvenience?

- Yes (1)
- Maybe (2)
- No (3)

Q51 Please indicate your reasons for responding yes to the question "Would you prefer in-person interaction with site staff during visits even if it required more travel and potential inconvenience?"

- Concerns about not feeling as comfortable asking questions as you would be if you were in person. (1)
- Concerns about of the interaction or communications might not be as good as if you were in person. (2)
- Concerns about the quality of the relationship you build with the clinical trial team. (3)
- Concerns about not being able to talk to the person on the clinical trial team you would like to (e.g., Investigator, clinical trial nurse, etc.) (4)
- Concerns about language barriers and potentially not having a caregiver present with you, not having interpreters available, etc. (5)
- Concerns about safety (6)
- Concerns about using technology (7)
- Concerns about privacy (either because of using technology or due to being in your own home) (8)

Q52 Please use the space below to let us know if you have any other concerns not listed above.

________________________________________________________________

End of Block: Questions about Travel

Start of Block: Questions about Study Data Collection sites

Q53 If required for the clinical trial, where would you prefer routine and simple tests be done or samples taken? For example, a routine test may be a blood pressure measurement or an x-ray or ultrasound. Samples might include taking blood, providing urine, etc. (check all that apply).

- At a study site (1)
- At your home (meaning that someone would be sent to your home to collect samples or do tests) (2)
- At a private lab close to home (3)
- No preference (4)

Q55 Please tell us your preference if it is not provided in the choices above.

________________________________________________________________

Q56 Please indicate your reason(s) for responding that you would prefer routine and simple tests be done or samples taken at a study site or combination. (check all that apply).

- Concerns about of the interaction or communications (1)
- Concerns about the quality of the relationship you build with the clinical trial team (2)
- Concerns about safety (3)
- Concerns about privacy (either because of using technology or due to being in your own home) (4)
- Concerns about not being able to talk to the person on the clinical trial team you would like to (e.g., Investigator, clinical trial nurse, etc.) (5)
- Concerns about language barriers and potentially not having a caregiver present with you, not having interpreters available, etc. (6)
- Concerns about the quality of the sample (e.g. staff at remote sites not being as knowledgeable about the study as site staff, etc.) (7)

Q57 Please use the space below to let us know if you have any other concerns not listed above.

________________________________________________________________

Q58 Please indicate your reason(s) for responding that you would prefer routine and simple tests be done or samples be taken at your home or at a private lab close to home. (check all that apply).

- Concerns about travel time to study site (1)
- Concerns about travel cost (2)
- Concerns about an unknown location/process (3)

Q59 Please use the space below to let us know if you have any other concerns not listed above.

________________________________________________________________

End of Block: Questions about Study Data Collection sites

Start of Block: Questions about the Patient Diary

Q60 A patient diary is a tool used in a clinical trial where participants record symptoms and information related to the intervention in the trial. Would you prefer keeping a patient diary for the clinical trial using:

- Typed or voice technology, such as a web-based interface or an app on your own computer or your own phone (1)
- Typed or voice technology on a device provided to you by the study team (2)
- Paper (3)
- No preference (4)

Q61 If you prefer another means of recording information please provide them in the space below.

________________________________________________________________

Q62 You chose that you would prefer a technology-based option, please indicate which of the following would be helpful: (check all that apply).

- Provision of clear instructions of how to use the technology (1)
- Access to IT support (2)
- No requirement to login or have a password when you use the technology (3)
- Having wifi or internet or data capabilities provided for its use (4)
- Knowing that your account would be deleted at the end of the study (e.g., for specific apps) (5)

Q63 Please let us know if anything else might be helpful  in the space below.

________________________________________________________________

End of Block: Questions about the Patient Diary

Start of Block: Questions about Home Delivery of Study materials

Q70 Please rank the following list of concerns related to having an investigational product (which might be a drug, device, or other intervention) delivered to your home from most to least. Use your mouse to drag your selections and place them in your preferred order. If you are using a phone or tablet use your finger.

______ Instructions or training provided to you by someone visiting your home. (1)

______ Instructions or training provided to you by someone via technology. (2)

______ Privacy with respect to the delivery. (3)

______ Having different people deliver product to you each time. (4)

______ Having others in your home (e.g. as it relates to safety and privacy). (5)

______ Delivery overall. (6)

______ A timeframe related to delivery. (7)

______ Security of the delivery (e.g. worried about the product being being stolen). (8)

Q71 Please let us know if you have any other concerns not mentioned above.

________________________________________________________________

End of Block: Questions about Home Delivery of Study materials

Start of Block: Questions about Accessibility

Q82 Do you think decentralized options for clinical trial participation could potentially make clinical trials more accessible to all Canadians (e.g. in rural and urban locations; for parents, caregivers? Etc.)?

- No (1)
- Unsure (2)
- Yes (3)

Q83 Do you think decentralized options for clinical trial participation could potentially increase the diversity of people who could participate in clinical trials?

- No (1)
- Unsure (2)
- Yes (3)

End of Block: Questions about Accessibility

Start of Block: Questions about entering information from home

Q64 If you participated in a clinical trial where you entered information for the trial from home how concerned would you be about the following:

Q65 Providing your information via technology?

- Not at all concerned (1)
- Slightly concerned (2)
- Somewhat concerned (3)
- Moderately concerned (4)
- Extremely concerned (5)

Q66 Your records being accessed by someone outside of the clinical trial team (called a ‘third’ party)?

- Not at all concerned (1)
- Slightly concerned (2)
- Somewhat concerned (3)
- Moderately concerned (4)
- Extremely concerned (5)

| Page Break |  |
| --- | --- |

Q67 Privacy of shared data to or from the clinical trial team (e.g. encryption of data or data being securely stored and/or transmitted)?

- Not at all concerned (1)
- Slightly concerned (2)
- Somewhat concerned (3)
- Moderately concerned (4)
- Extremely concerned (5)

Q68 Please let us know if you have any other concerns not mentioned above.

________________________________________________________________

End of Block: Questions about entering information from home
