# Supplementary material for: Patient and Public Perceptions in Canada About Decentralized and Hybrid Clinical Trials: “It’s About Time we Bring Trials to People”
Source: Ther Innov Regul Sci. 2024 Jun 21;58(5):965–77. doi: 10.1007/s43441-024-00665-y (PMC11335844; doi:10.1007/s43441-024-00665-y)
Supplement: Supplementary file 4 — Supplementary Material 4 - Results of questions related to data collection, keeping a patient diary, home delivery of study materials, and accessibility [file 43441_2024_665_MOESM4_ESM.docx]

**Supplementary Material 2**

| **Questions about study data collection sites** |  |
| --- | --- |
|  |  |
| **Question and Response Options** | **Response** |
| **If required for the clinical trial, where would you prefer routine and simple tests be done or samples taken?** | Count (%) |
| At a private lab close to home | 127 (72.6%) |
| At your home | 60 (34.3%) |
| At a study site | 25 (14.3%) |
| no preference | 20 (11.4%) |
|  |  |
| **Please indicate your reason(s) for responding that you would prefer routine and simple tests be done or samples be taken at your home or at a private lab close to home (check all that apply).** | Checked (%) |
| Concerns about travel time to study site | 146 (91.3%) |
| Concerns about travel cost | 84 (52.5%) |
| Concerns about an unknown location/process | 42 (26.3%) |
|  |  |
| **Please indicate your reason(s) for responding that you would prefer routine and simple tests be done or samples taken at a study site or combination (check all that apply).** | Checked (%) |
| Concerns about the quality of the sample (e.g., staff at remote sites not being as knowledgeable about the study as site staff, etc.) | 65 (59.1%) |
| Concerns about not being able to talk to the person on the clinical trial team you would like to (e.g., Investigator, clinical trial nurse, etc.) | 51 (46.4%) |
| Concerns about privacy (either because of using technology or due to being in your own home) | 35 (31.8%) |
| Concerns about the quality of the relationship you build with the clinical trial team | 31 (28.2%) |
| Concerns about safety | 29 (26.4%) |
| Concerns about interaction or communications | 23 (20.9%) |
| Concerns about language barriers and potentially not having a caregiver present with you, not having interpreters available, etc. | 6 (5.5%) |
|  |  |

**Questions about the patient diary**

| **Question** | **Response** |
| --- | --- |
| **A patient diary is a tool used in a clinical trial where participants record symptoms and information related to the intervention in the trial. Would you prefer keeping a patient diary for the clinical trial using:** | Count (%) |
| Typed or voice technology, such as a web-based interface or an app on your own computer or your own | 113 (64.6%) |
| Typed or voice technology on a device provided to you by the study team | 62 (35.4%) |
| Paper | 15 (8.75) |
| No Preference | 42 (24.3%) |
|  |  |
| **You chose that you would prefer a technology-based option, please indicate which of the following would be helpful (check all that apply):** | Checked (%) |
| Provision of clear instructions of how to use the technology | 101 (90.2%) |
| Access to IT support | 81 (72.3%) |
| Knowing that your account would be deleted at the end of the study | 61 (54.5%) |
| No requirement to login or have a password when you use the technology | 29 (25.9%) |
| Having wifi or internet or data capabilities provided for its use | 19 (17.0%) |

**Questions about home delivery of study materials**

| **Question** | **Response** |
| --- | --- |
| **Please rank the following list of concerns related to having an investigational product (which might be a drug, device, or other intervention) delivered to your home from most to least. Use your mouse to drag your selections and place them in your preferred order. If you are using a phone or tablet use your finger.** | Rank average* |
| Instructions or training provided to you by someone visiting your home. | 3.16 |
| Instructions or training provided to you by someone via technology | 3.40 |
| A timeframe related to delivery | 4.22 |
| Privacy with respect to the delivery | 4.60 |
| Security of the delivery | 4.84 |
| Having others in your home | 5.02 |
| Delivery overall | 5.25 |
| Having different people deliver product to you each time | 5.34 |

*A low average indicates increased order of importance. For example, an average score of 1 would represent the greatest possible order of importance whereas an average score of 5 would represent the least possible order of importance.

**Questions about accessibility**

| **Question and Response Options** | **Response** |
| --- | --- |
| **Do you think decentralized options for clinical trial participation could potentially make clinical trials more accessible to all Canadians (e.g., in rural and urban locations; for parents, caregivers?)** |  |
| Yes | 152 (91.0%) |
| No | 3 (1.8%) |
| Unsure | 12 (7.2%) |
|  |  |
| **Do you think decentralized options for clinical trial participation could potentially increase the diversity of people who could participate in clinical trials?** |  |
| Yes | 143 (85.1%) |
| No | 6 (3.6%) |
| Unsure | 19 (11.3%) |
